# The interplay between mobilome and resistome in *Staphylococcus aureus*

**DOI:** 10.1128/mbio.02428-24

**Published:** 2024-09-17

**Authors:** Rachel Contarin, Antoine Drapeau, Pauline François, Jean-Yves Madec, Marisa Haenni, Emilie Dordet-Frisoni

**Affiliations:** 1 INTHERES, Université de Toulouse, INRAE, ENVT, Toulouse, France; 2 Anses—Université de Lyon, Unité Antibiorésistance et Virulence Bactériennes, Lyon, France; University of Pretoria, Pretoria, Gauteng, South Africa

**Keywords:** *Staphylococcus aureus*, mobile genetic elements, resistome, horizontal gene transfer, antimicrobial resistance, plasmids, mobilome, transposons

## Abstract

Antibiotic resistance genes (ARGs) in *Staphylococcus aureus* can disseminate vertically through successful clones, but also horizontally through the transfer of genes conveyed by mobile genetic elements (MGEs). Even though underexplored, MGE/ARG associations in *S. aureus* favor the emergence of multidrug-resistant clones, which are challenging therapeutic success in both human and animal health. This study investigated the interplay between the mobilome and the resistome of more than 10,000 *S*. *aureus* genomes from human and animal origin. The analysis revealed a remarkable diversity of MGEs and ARGs, with plasmids and transposons being the main carriers of ARGs. Numerous MGE/ARG associations were identified, suggesting that MGEs play a critical role in the dissemination of resistance. A high degree of similarity was observed in MGE/ARG associations between human and animal isolates, highlighting the potential for unrestricted spread of ARGs between hosts. Our results showed that in parallel to clonal expansion, MGEs and their associated ARGs can spread across different strain types sequence types (STs), favoring the evolution of these clones and their adaptation in selective environments. The high variability of MGE/ARG associations within individual STs and their spread across several STs highlight the crucial role of MGEs in shaping the *S. aureus* resistome. Overall, this study provides valuable insights into the complex interplay between MGEs and ARGs in *S. aureus*, emphasizing the need to elucidate the mechanisms governing the epidemic success of MGEs, particularly those implicated in ARG transfer.

**IMPORTANCE:**

The research presented in this article highlights the importance of understanding the interactions between mobile genetic elements (MGEs) and antibiotic resistance genes (ARGs) carried by *Staphylococcus aureus*, a versatile bacterium that can be both a harmless commensal and a dangerous pathogen for humans and animals. *S. aureus* has a great capacity to acquire and disseminate ARGs, enabling efficient adaption to various environmental or clinical conditions. By analyzing a large data set of *S. aureus* genomes, we highlighted the substantial role of MGEs, particularly plasmids and transposons, in disseminating ARGs within and between *S. aureus* populations, bypassing host barriers. Given that multidrug-resistant *S. aureus* strains are classified as a high-priority pathogen by global health organizations, this knowledge is crucial for understanding the complex dynamics of transmission of antibiotic resistance in this species.

## INTRODUCTION

*Staphylococcus aureu*s is a versatile bacterium that can be a commensal microorganism and a lethal pathogen for both humans and animals. The large range of symptoms caused by *S. aureus* is the result of the intricate interplay between complex factors, including host health status, genetic composition of the *S. aureus* strain, and the site of infection (1–3). The overuse and misuse of antibiotics in both human healthcare and veterinary medicine have promoted the emergence of numerous antibiotic-resistance genes (ARGs) and the dissemination of multidrug-resistant clones (4). As a result, this species has been classified by the World Health Organization (WHO) as high-priority for the development of new antibiotic targets, and by the World Organization for Animal Health (WOAH) on the surveillance list of multidrug-resistant pathogens. Studies on antimicrobial resistance (AMR) in *S. aureus* have primarily focused on methicillin-resistance, due to the presence of the *mecA/mecC* genes, which confer resistance to all beta-lactam antibiotics. Consequently, *S. aureus* strains are commonly classified as methicillin-resistant (MRSA) or methicillin-susceptible isolates (5). However, *S. aureus* can acquire multiple additional ARGs conferring resistance to all known antibiotic families, shaping the resistome of each isolate (6). In 2018, a review drew up an inventory of 46 ARGs shared between *S. aureus* isolates of human and animal origin, suggesting potential interspecies transmissions (7).

In contrast to a highly clonal core genome, up to 25% of the *S. aureus* genome is composed of accessory genes, primarily encompassing mobile genetic elements (MGEs) that contribute significantly to the adaptability and survival of these bacteria in various environmental conditions by spreading ARGs through horizontal gene transfer (HGT) (8, 9). MGEs are DNA fragments that can promote intra- or intercellular transfer of genetic material, and play a crucial role in the dissemination of ARGs. The staphylococcal mobilome (set of MGEs) exhibits a remarkable variability (8, 10–13). *S. aureus* MGEs include (i) plasmids (extrachromosomal genetic elements that can be conjugative, mobilizable, or non-mobilizable in *S. aureus*) (12), (ii) integrative and conjugative elements (ICEs) (MGEs that integrate into the bacterial chromosome and have the potential to excise and transfer themselves to other bacteria via conjugation) (14, 15), (iii) insertion sequences (IS) and transposons (Tn) (chromosomally-integrated MGEs that are often associated with other MGEs, in single or multiple copies) (13), (iv) prophages (MGEs usually integrated into the host’s chromosome, since they can repress the phage’s lytic functions) (16), and (v) genomic islands (such as the staphylococcal chromosomal cassettes (SCC*mec*) specific to *mec*-carrying staphylococci) (13).

It is usually admitted that *S. aureus* disseminates through clonal expansion, spreading resistance genes vertically through waves of successful lineages. While the role of MGEs, particularly plasmids, has been extensively studied in Enterobacterales, few studies have addressed the diverse MGEs and associated ARGs carried by *S. aureus* (8, 9, 13, 17). Likewise, no global-scale analyses have been conducted to localize ARGs in human and animal *S. aureus* genomes from worldwide. To fill in this knowledge gap, this study aimed at (i) identifying the most prevalent MGEs and ARGs in 10,063 *S*. *aureus* genomes from the NCBI public database, (ii) highlighting MGE/ARG associations, and (iii) evaluating the role of MGEs in the emergence and spread of antibiotic resistance in *S. aureus*.

## RESULTS

### Diversity of human and animal *S. aureus* genomes

**Fig 1:**
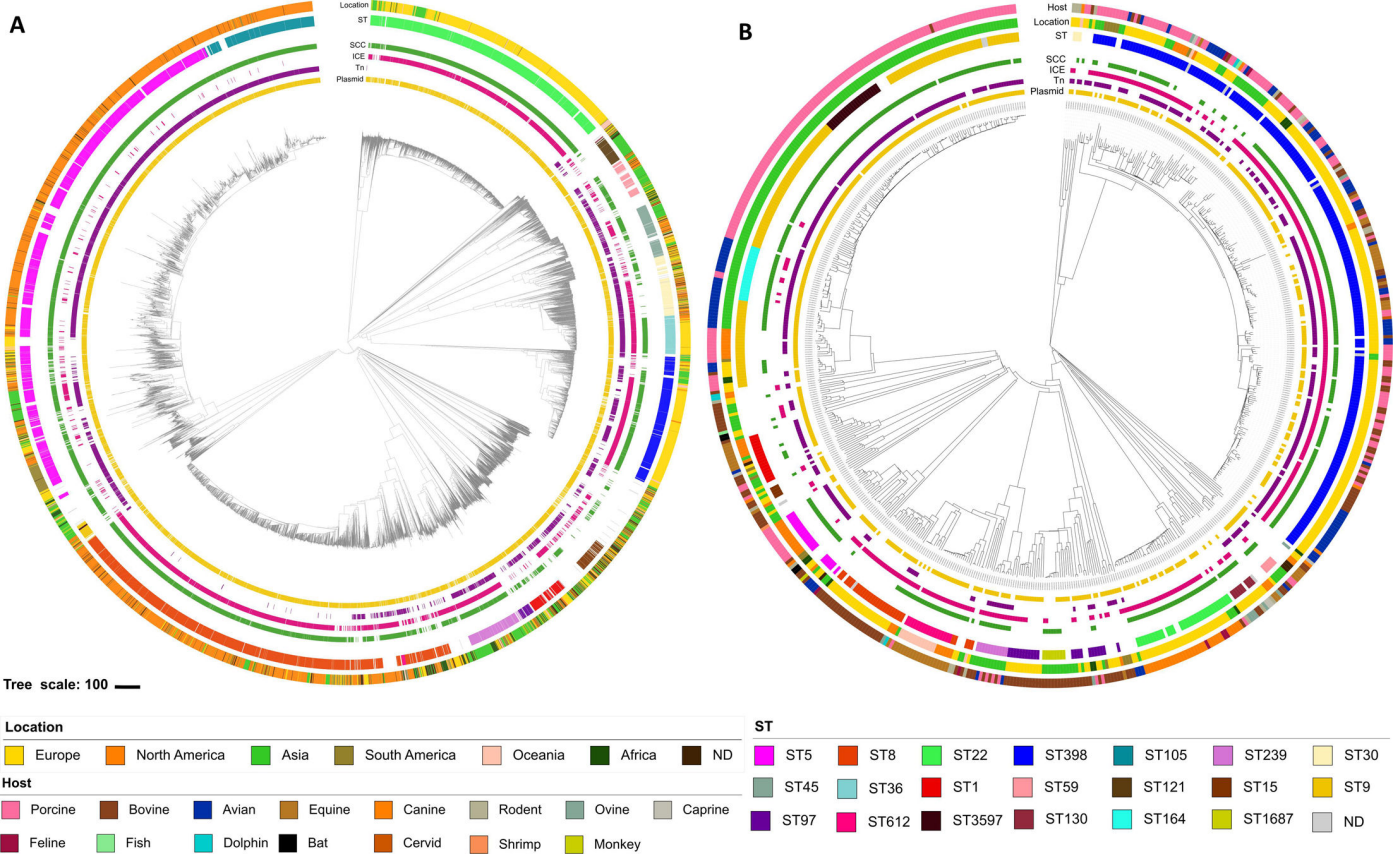
Genomic diversity of Hm-SA (**A**) and An-SA (**B**) origin. The phylogenetic trees represent 9,408 Hm-SA and 655 An-SA. This tree based on cgMLST distance was constructed using the Neighbor-Joining method and visualized using iTOL v6 (https://itol.embl.de). From outer circle to inner circle, host (only for An-SA), location, ST, SCC (green), ICE (pink), Tn (purple), and plasmids (yellow) were represented. The correspondence between colors and each type of host, location, or ST is indicated below the figure. Only STs with a frequency of at least 1% in Hm-SA or in An-SA were represented. Genomes with no associated ST and/or no location were annotated as “not determined” (ND). As they were present in up to 90% of the analyzed genomes, IS and prophages were not represented.

A total of 9,408 human and 655 animal *S. aureus* genomes were analyzed. The animal *S. aureus* genomes came from 15 different hosts, mainly livestock (527/655, Table S1) but also from companion animals and wildlife. Data originated from all continents, with a significant proportion of *S. aureus* of human origin (Hm-SA) isolated from North America and Europe, and of *S. aureus* of animal origin (An-SA) isolated from Europe and Asia (Fig. 1; Table S1). The core genome of all *S. aureus* strains consisted of 1,861 genes, including three genes unique to Hm-SA and 45 genes unique to An-SA (Table S2). A total of 433 distinct sequence types (STs) were identified, but only 21 of them were found in at least >1% Hm-SA or An-SA. Among the 433 STs, 362 (three present in >1% of Hm-SA) were unique to Hm-SA, 34 (two present in >1% of An-SA) were unique to An-SA*,* and 37 (16 present in >1% of Hm-SA and An-SA) were shared between both groups, highlighting the specificity of STs according to the human or animal origin (Fig. S1A).

The most frequently identified STs were (i) ST5 (2,138/9,408) and ST8 (*n* = 1,719) in Hm-SA and (ii) the two major livestock-associated ST398 (216/655) and ST9 (*n* = 125) in An-SA (Fig. 1; Fig. S1B) Some STs had geographic niches (ST5/ST8 in North America, ST22/ST398 in Europe, and ST9 in Asia), while others (such as ST1 and ST45) were present worldwide (Fig. S2A and B).

### Mobile genetic elements arsenal in *S. aureus* genomes

Analysis of accessory genomes highlighted a remarkable diversity of MGEs including ICEs, transposons, and plasmids regardless of their human or animal origin (Fig. 1; Table S1). A total of 114,845 different MGEs were detected (Table 1; Table S1). The most prevalent ones were IS, followed by prophages, plasmids, and SCC*mec* (Table 1). Transposons, ICEs, and composite transposons (CTn) were the least common MGEs detected in *S. aureus* genomes. Transposons and ICEs were similar whether they came from humans or animals, contrarily to IS and plasmids. These elements were partially specific according to their origin since only 33% of IS (21/64 IS families identified) and 23% of plasmids (69/297 plasmid families) were shared between Hm-SA and An-SA genomes (Table 1).

**TABLE 1:** Prevalence of MGEs identified in Hm-SA and An-SA origin

Type of MGE	Plasmid		Transposon		ICE		SCC		Phage		IS		CTn		All MGEs		All MGEs (except IS)	
Origin	Hm	An	Hm	An	Hm	An	Hm	An	Hm	An	Hm	An	Hm	An	Hm	An	Hm	An
Number of MGEs identified	12,084	929	6,581	499	5,287	436	7,518	471	19,124	867	52,204	2,873	5,585	387	108,383	6,462	56,179	3,589
Mean MGEs per genome	1.5	1.7	1.4	1.2	1.1	1.2	1	1	2.1	1.6	5.6	4.5	1.7	1.9	11.5	9.9	6.0	5.5
Maximum of MGEs per genome	8	6	7	3	4	3	1	1	6	5	58	41	17	11	78	57	23	16
Strains containing MGEs (% tot genomes)^*a*^	8,064 (86%)	532 (81%)	4,707 (50%)	420 (64%)	4,751 (50%)	363 (55%)	7,518 (80%)	471 (72%)	9,090 (97%)	554 (85%)	9,286 (99%)	636 (97%)	3,368 (36%)	206 (31%)	9,408 (100%)	655 (100%)	9,408 (100%)	655 (100%)
MGEs specific to Hm/An genomes	203	25	2	0	0	0	12	1	30	4	35	8	10	2	292	40	257	32
MGEs shared between Hm and An genomes/total types of MGE	69/297		9/11		3/3		18/31		51/85		21/64		10/22		181/513		160/449	

^*a*^ Number of MGE/total number of genomes *n* = 9,408 for Hm-SA and *n* = 655 for An-SA (in %)*.*

The MGE content varied depending on STs. Plasmids, IS, and SCC were present in all major STs (found in >1% of isolates), regardless of their origin. However, specific MGEs could be absent from certain STs, such as transposons that were not identified in ST22 genomes (Fig. S3A and B). The mobilome also varied among isolates of the same ST, particularly in their plasmid and transposon content, as observed for Hm-SA ST8 and An-SA ST398 (Fig. 1; Table S1).

**Fig 2:**
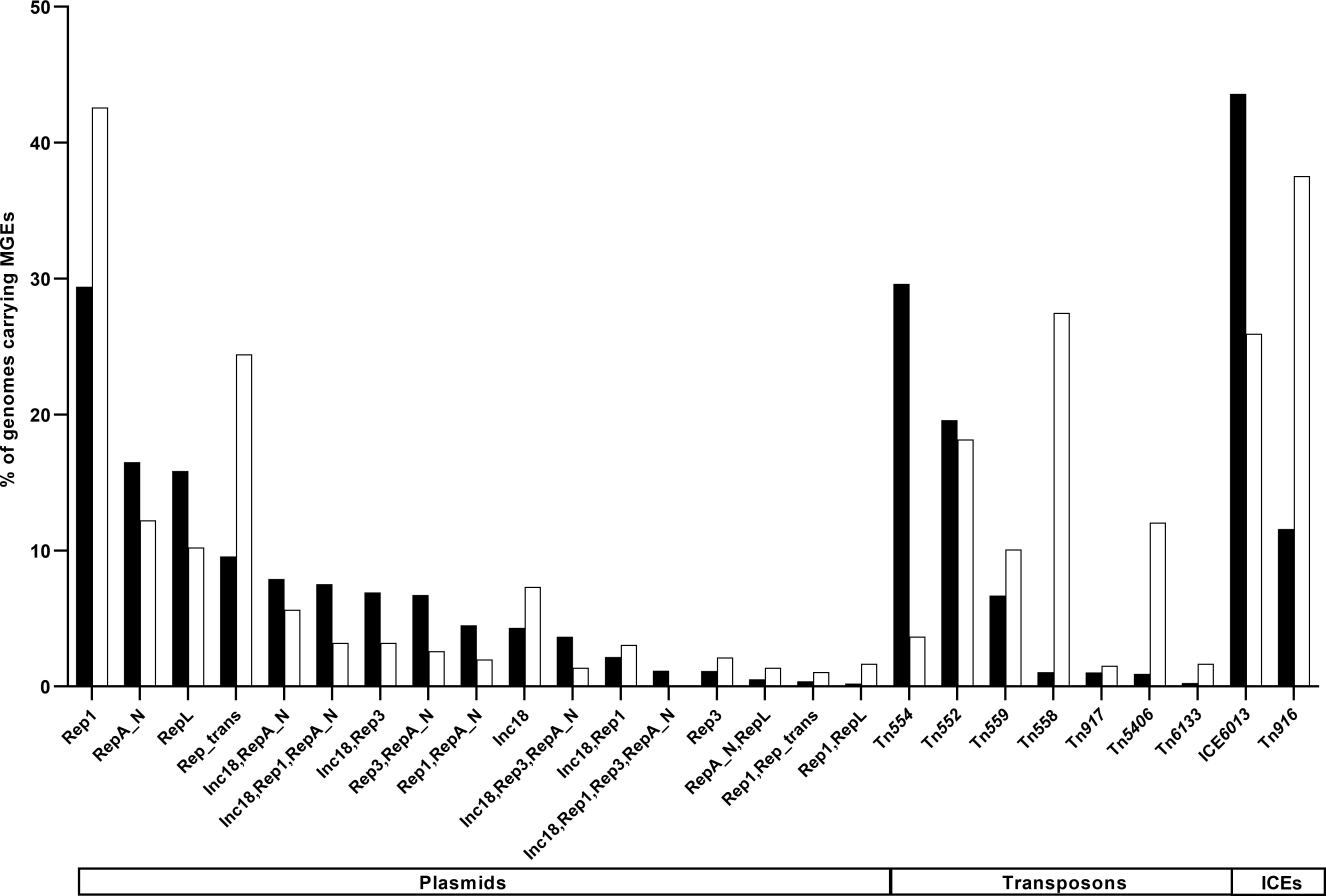
Diversity and occurrence of the most prevalent MGE families (plasmids, transposons, and ICEs) among Hm-SA (black) and An-SA (white) origin. Bar chart of the percentage of genomes carrying MGEs corresponding to the number of Hm-SA or An-SA genomes harboring at least one MGE over the total number of Hm-SA or An-SA genomes analyzed (Hm-SA *n* = 9,408 and An-SA *n* = 655). Only MGEs identified in at least 1% of Hm-SA or An-SA genomes are represented in the x-axis.

**Fig 3:**
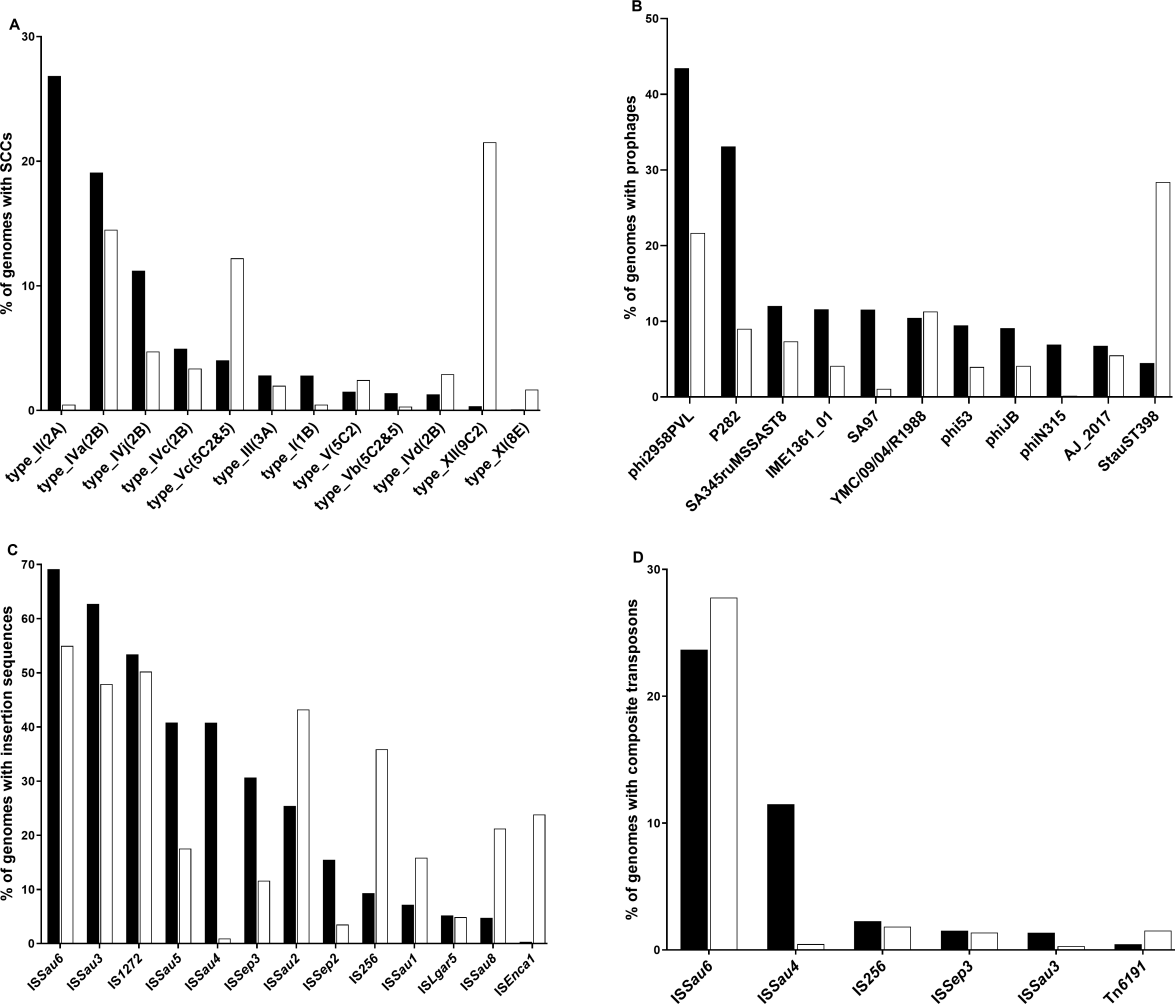
Occurrence and families of SCCs (**A**), prophages (**B**), IS (**C**), and CTn (**D**) detected in Hm-SA (black) and An-SA (white) genomes. Bar charts of the percentage of genomes carrying MGEs correspond to the number of Hm-SA or An-SA genomes carrying at least one MGE over the total number of Hm-SA or An-SA genomes analyzed (Hm-SA *n* = 9,408 and An-SA *n* = 655). For SCC and CTn, only MGEs identified in at least 1% of Hm-SA or An-SA genomes were represented. For prophages and IS only MGEs identified in at least 5% of Hm-SA or An-SA genomes were depicted.

Different families were identified for each of the MGE types (Fig. 2; Table S1). Among plasmids, Rep1 was predominant in both Hm-SA (2,768/9,408; 29%) and An-SA (279/655; 43%) genomes, while others segregated depending on the origin, like RepA_N and RepL in Hm-SA (16%) versus Rep_trans in An-SA (24%) (Fig. 2). Plasmids also frequently harbored multiple *rep* genes (4,490/12,084; 37% plasmids in Hm-SA and 213/929; 23% in An-SA). For transposons, the Tn*554* family was frequent in Hm-SA (30%), the Tn*558* family in An-SA (27%), and the Tn*552* was equally distributed in both (20%) (Fig. 2). Concerning ICEs, the ICE*6013* family prevailed in Hm-SA (44%, versus 26% in An-SA; Fisher’s exact test, *P*-value < 0.0001), whereas the Tn*916* family (originally named Tn, but considered as an ICE) was more frequent in An-SA (38%) than in Hm-SA (12%; *P*-value < 0.0001) (Fig. 2). For SCC*mec*, type II(2A) and type IVa(2B) were frequent in Hm-SA (27% and 19%, respectively), while type XII(9C2) was the most prevalent (21.5%) in An-SA (Fig. 3A). Among prophages, the most common were phi2958PVL and P282 families, present in more than 33% of Hm-SA genomes, while the prophage StauST398 was the most abundant in An-SA (28%) (Fig. 3B). For IS, the most common families were IS*Sau6*, IS*Sau3,* and IS*1272*, regardless of the origin (Fig. 3C). IS*Sau4* was frequent in Hm-SA (41%) and almost absent in An-SA (0.9%) (Fig. 3C). IS*256* was abundant in An-SA (36%) but rarely found in Hm-SA (9%) (Fig. 3C). Finally, as observed for ISs, the CTn containing IS*Sau6* was the most prevalent, identified in >23% of *S. aureus* genomes, regardless of their origin (Fig. 3D). The CTn with IS*Sau4* was abundant in Hm-SA genomes (11.5%) but rare in An-SA genomes (0.5%).

### *S. aureus* antibiotic resistance genes content

**Fig 4:**
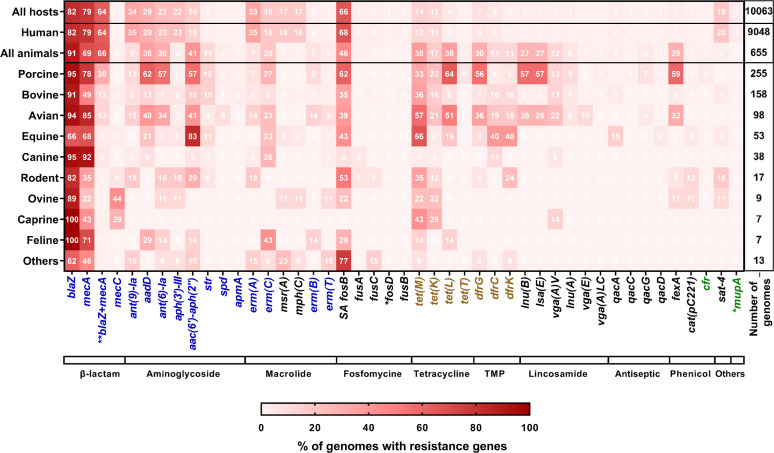
Occurrence of ARGs in *S. aureus* genomes. *S. aureus* hosts were indicated on the left side of the heatmap, detected ARGs at the bottom, and the total number of genomes analyzed for each host on the right side. Hosts grouped under the label “others” correspond to dolphin (*n* = 4), bat (*n* = 4), fish (*n* = 2), cervid (*n* = 1), shrimp (*n* = 1), and monkey (*n* = 1). The bar at the bottom of the heatmap corresponds to the families of antibiotics with TMP for trimethoprim and “others” for nucleoside (*sat-4*) and mupirocin (*mupA*) families. The percentages of genomes containing ARGs were obtained by dividing the number of genomes possessing the resistance gene by the total number of genomes on the top right of the heatmap. These percentages were represented by different shades of red color, as shown at the bottom of the heatmap. Only ARGs with a frequency of at least 0.1% in Hm-SA or in An-SA, were represented. Genes exclusively present in Hm-SA are indicated by an asterisk (*). The double asterisk (**) indicates genomes containing both *blaZ* and *mecA* genes, with the percentage calculated as the number of such genomes divided by the total number of genomes with either *blaZ* or *mecA*. Highlighted genes are categorized as follows: (i) those conferring resistance to antibiotics listed as “human-use only,” HICIA and CIA in the WHO list (green), (ii) those listed as veterinary CIA in the WOAH list (brown), and (iii) those included in both lists (blue).

A total of 52,447 ARG sequences were identified from Hm-SA genomes, and 4,370 from An-SA genomes, and each genome harbored an average of five (Hm-SA) or six (An-SA) different ARGs per genome, with a maximum of 17 per Hm-SA and 15 per An-SA genome (Table S1). Seventy-eight different ARGs were identified, most having a worldwide distribution (Fig. S2C and D). Fifty of them were shared by both Hm-SA and An-SA genomes, 27 were unique to Hm-SA, and one (*optrA*) was unique to An-SA (Fig. 4; Table S1). Among the 44 genes identified in >1% of genomes, 15 conferred resistance to critically important antibiotics (CIA) as defined by the WHO and WOAH (18, 19) (Fig. 4; Table S1). In Hm-SA, some ARGs were frequently collocated, such as *ant (9)-Ia* and *erm(A)* (3,251/3,334 genomes with at least one of these two genes), *ant (6)-Ia* and *aph(3')-III* (2,024/2,296), and *mph(C*) and *msr(A)* (1,619/1,838) (Fig. S4). Similarly, in An-SA isolates, the *lnu*(B) gene was found in tandem with the *lsa*(E) gene in 175/176 genomes presenting at least one of these two genes (Fig. S4).

The *blaZ* and *mecA* genes, conferring resistance to beta-lactam antibiotics, were commonly found regardless of their origin and often co-harbored (5,913/9,245 genomes with at least one of the two genes in Hm-SA and 417/630 in An-SA) (Fig. 4; Fig. S3 and S4). The *ant (9)-Ia* and *aph(3')-III* (aminoglycosides) and *erm(A)* (macrolides) genes were more common in Hm-SA, while the *aac(6')-aph(2'')* (aminoglycoside), *tet(M*) and *tet(L)* (tetracyclines), *dfrG* (trimethoprim)*, lnu(B) and lsa(E)* (lincosamides) and *fexA* (phenicol) genes were each present in >25% of An-SA genomes (Fig. 4). Of note, the *aac(6')-aph(2''*) gene was found in 83% (44/53) of the equine genomes, while it was present in <60% of those from other animal hosts (Fig. 4). Among animals, pigs presented *S. aureus* genomes with the largest number of resistance genes, with 11 genes belonging to seven different families present in more than 40% of the genomes (Fig. 4). In contrast, in bovine *S. aureus*, only *blaZ* and *mecA* were present in more than 40% of the genomes (Fig. 4). Certain ARGs exhibited restricted distribution patterns, as *vga(E*) present only in human-derived ST398 *S. aureus* strains or *vga(A)LC* and *apmA* identified in animal-derived ST398/ST9 strains (Fig. S3C and D). The ARG/ST associations were origin-specific, except for *str*, *spd*, *ampA*, *erm(T*), *dfrK,* and *vga(E*), which were predominantly (>80%) identified in ST398 genomes in both Hm-SA and An-SA (Fig. S3C and D).

### Association of mobile genetic elements with antibiotic resistance genes in *S. aureus*

A Pearson correlation analysis revealed a strong positive correlation between the number of ARGs and the number of MGEs (excluding isolated IS elements, which do not mobilize ARGs) in *S. aureus* genomes (r = 0.57, *P* < 0.0001, r = 0.39, *P* < 0.0001, for Hm-SA and An-SA, respectively). This correlation was particularly high for transposons, with r-values of 0.45 (*P* < 0.0001) for Hm-SA and 0.60 (*P* < 0.0001) for An-SA strains, but also for SCC elements (r = 0.45, *P* < 0.0001; r = 0.48, *P* < 0.0001) and plasmids (r = 0.24, *P* < 0.0001; r = 0.46, *P* < 0.0001).

**Fig 5:**
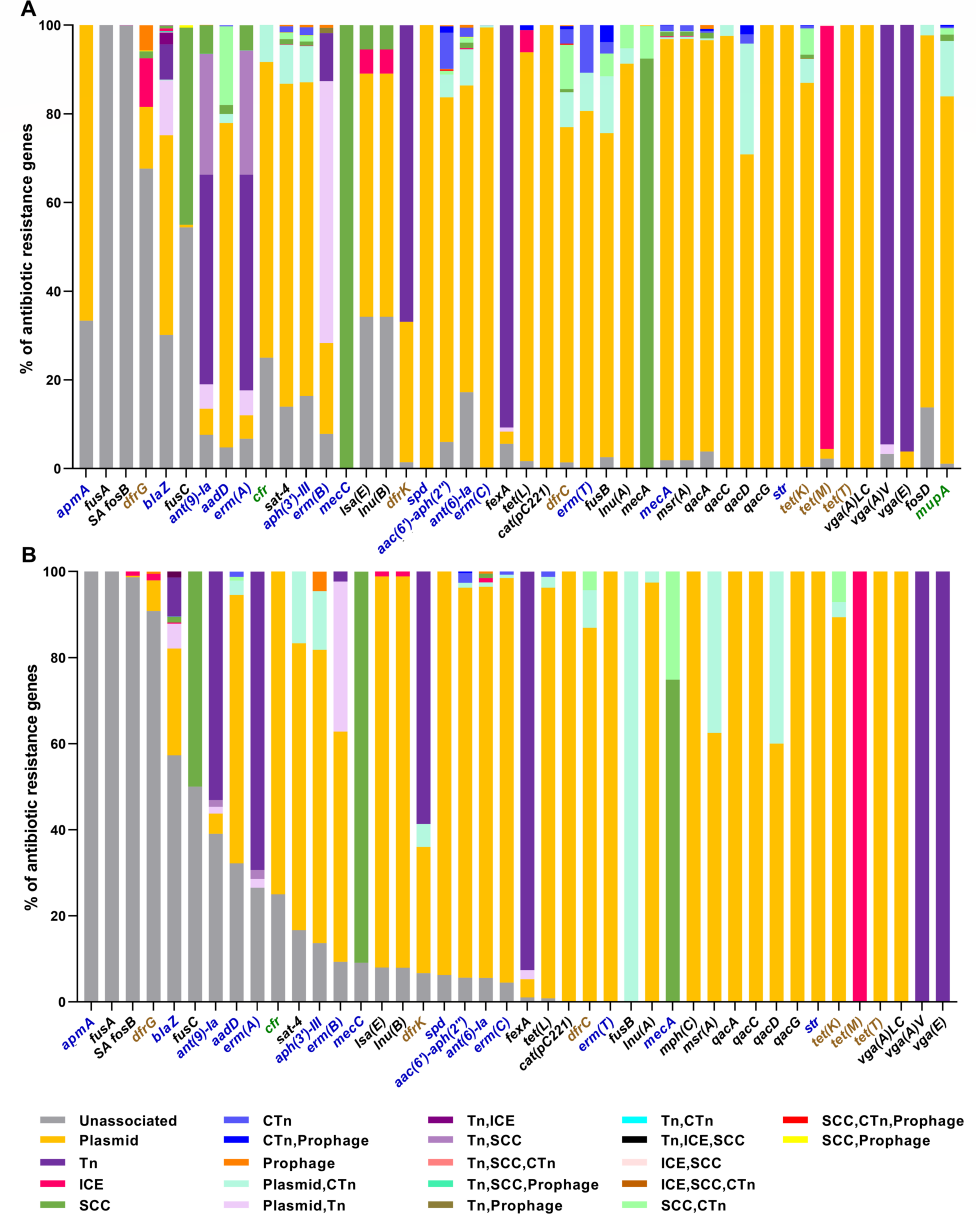
Associations of ARGs and MGEs in Hm-SA (**A**) and An-SA (**B**) origin. Bar chart representing the percentage of associations between ARGs and MGEs, with a color assigned to each association and indicated in the legend. When an ARG was not associated, the percentage was represented in gray. Only ARGs with a frequency of at least 0.1% in Hm-SA or An-SA were depicted. The percentage of ARG corresponds to the number of ARG associated with MGE or a combination of MGEs divided by the total number of this ARG, identified in Hm-SA or An-SA. Highlighted genes are categorized as follows: (i) those conferring resistance to antibiotics listed as “human-use only,” HICIA and CIA in the WHO list (green), (ii) those listed as veterinary CIA in the WOAH list (brown), and (iii) those included in both lists (blue).

The majority of ARGs (40,911/52,447; 78% ARGs identified in Hm-SA, 3,266/4,370; 75% in An-SA) were associated with MGEs (Fig. 5; Table S3). Plasmids were the predominant MGE harboring ARGs (42% of ARGs identified in Hm-SA and 47% in An-SA), followed by SCC elements (22% in Hm-SA and 11% in An-SA) and transposons (18% in Hm-SA and 12% in An-SA) (Fig. 5; Table S3). MGEs can be nested like Russian dolls; for example plasmids or ICEs can harbor transposons (Fig. 5). A total of 21 and 12 MGE/ARG combinations were identified in Hm-SA and An-SA, respectively (Fig. 5). The *spd*, *cat(pC221*), *qacG*, *str*, *tet(T*), and *vga(A)LC* genes were exclusively found on plasmids, while *ant (9)-Ia*, *erm(A*), *fexA*, *vga(A)V,* and *vga(E*) were primarily associated with transposons, regardless of the *S. aureus* origin (Fig. 5). All antibiotic families were found associated with plasmids, while only tetracycline-resistance has never been found associated with transposons. Several genes were associated with more than one MGE, like *blaZ*, *erm(B*), and *dfrK* which can be carried by plasmids, transposons, or putative plasmid/transposon tandem (Fig. 5).

#### Plasmid/ARG associations

**Fig 6:**
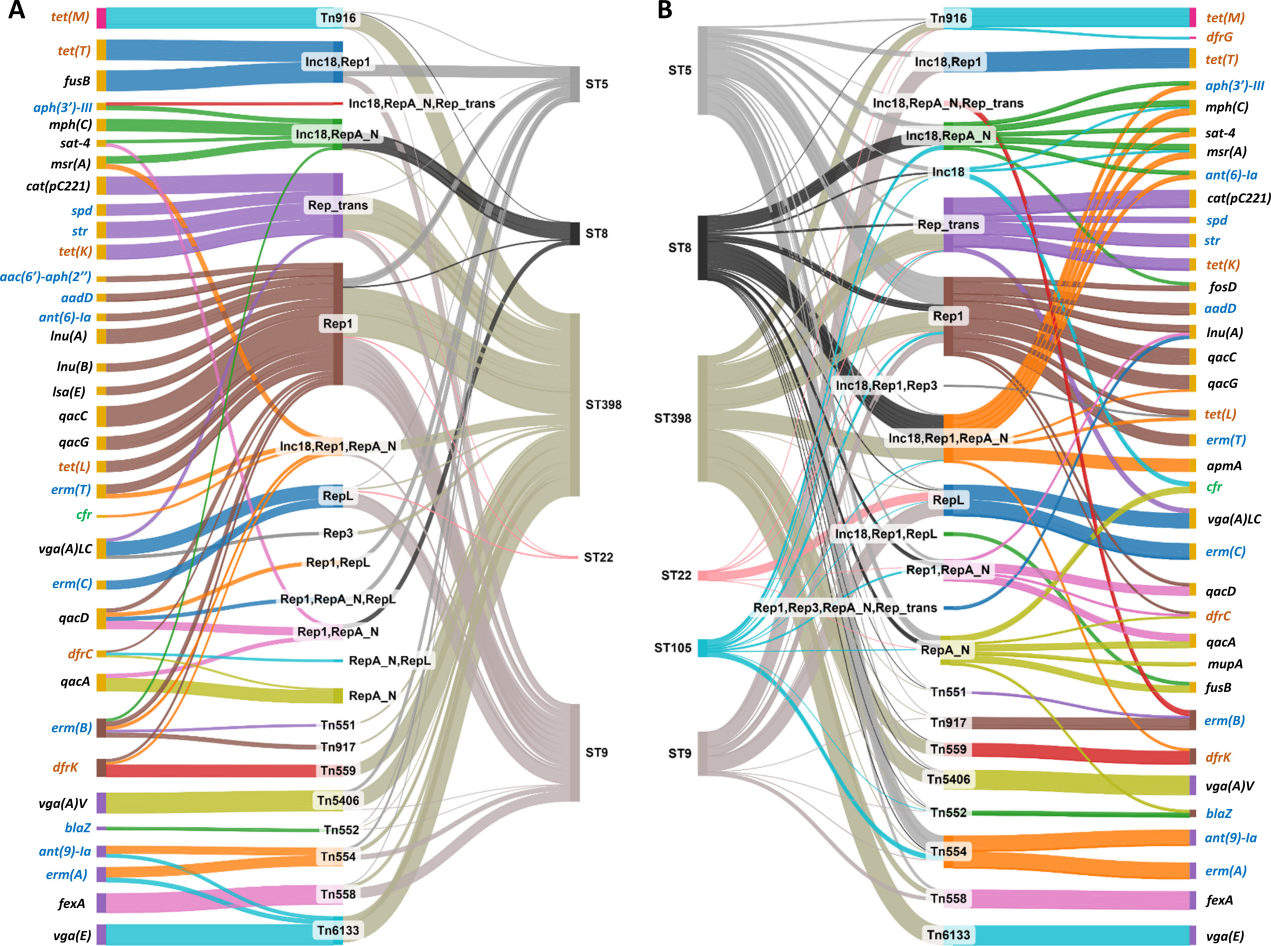
Associations of ARGs with plasmids, transposons, and ICEs in An-SA (**A**) and Hm-SA (**B**) genomes depending on ST. Each ARG, MGE, and ST was represented by a node linked by a flow when associated. The color of the ARG nodes corresponds to the type of MGE to which it is associated: pink for ICE, yellow for plasmid, purple for transposon, and brown for plasmid harboring transposon. The thickness of the flow is proportional to the percentage of ARGs associated with the MGE and the percentage of MGE associated with ST. This percentage is calculated by dividing the number of ARGs associated with the MGE by the total number of these ARGs identified in An-SA or Hm-SA genomes. Only ARGs with a frequency of at least 0.1% in Hm-SA or An-SA and association frequencies of MGE/ARG >10% were represented. For the MGE/ST associations, only the six most abundant STs in either Hm-SA or An-SA were represented. The maximum thickness of the flow corresponds to 100%, as demonstrated by Tn*916*/*tet(M*) for (**A**) and Inc18, Rep1/*tet(T*) for (**B**). Highlighted genes are categorized as follows: (i) those conferring resistance to antibiotics listed as “human-use only,” HICIA and CIA in the WHO list (green), (ii) those listed as veterinary CIA in the WOAH list (brown), and (iii) those included in both lists (blue). Sankey diagrams were created using SankeyMATIC (http://sankeymatic.com).

**Fig 7:**
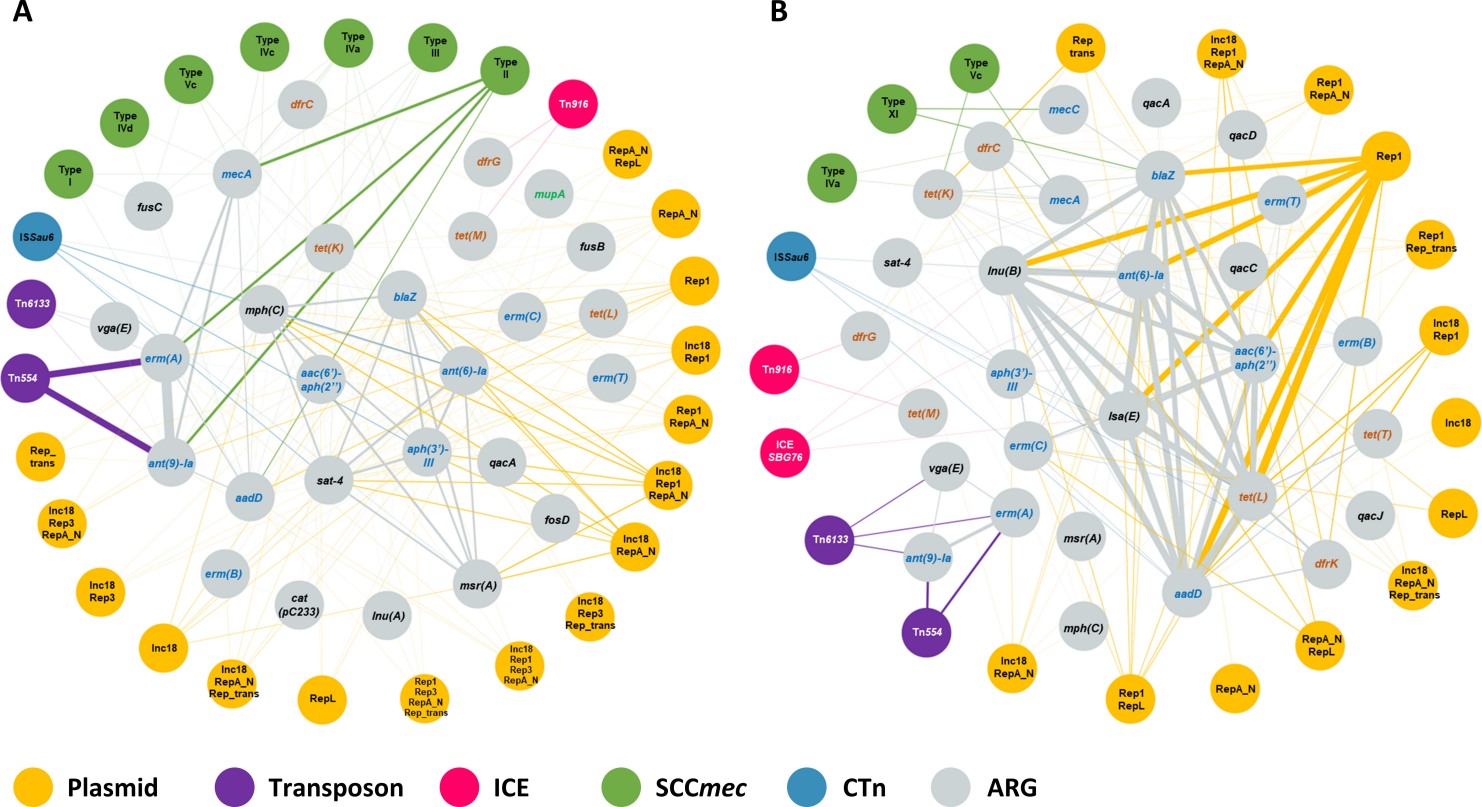
Associations between multiple ARGs and MGEs in Hm-SA (**A**) and An-SA (**B**) genomes. Associations were represented by links that indicate that ARGs were found within the same genome on the same MGE. The thickness of the line is proportional to the number of genomes carrying this co-occurrence. The thickest lines correspond to Rep1/*aac(6’)-aph(2’’)/aadD/ant (6)-Ia/erm(C)/lnu(B)/lsa(E)/tet(L*) association identified in 42 An-SA genomes and Tn*554*/*ant (9)-Ia*/*erm(A*) association in 3,643 Hm-SA genomes. Only associations found in at least 10 Hm-SA genomes or two An-SA genomes were shown. Highlighted genes are categorized as follows: (i) those conferring resistance to antibiotics listed as “human-use only,” HICIA and CIA in the WHO list (green), (ii) those listed as veterinary CIA in the WOAH list (brown), and (iii) those included in both lists (blue).

Each ARG was primarily associated with a specific Rep family (Fig. 6). Rep1 and RepA_N were the dominant plasmid families associated with ARGs, either alone or in combination with other Rep (496/619; 80% plasmid/ARG combinations in Hm-SA; 164/210; 78% in An-SA) (Fig. 6). Rep1/*aadD* (1,603/22,135; 7% ARGs associated with plasmid) in Hm-SA and Rep1/*tet(L*) (133/2,038; 6.5%) in An-SA were the most common. In Hm-SA, the two most frequent plasmid/multi-ARGs associations were Inc18/Rep1/RepA_N (401/3,210) and Inc18/RepA_N (*n* = 363), each associated with the same six ARGs [*ant (6)-Ia*, *aph(3’)-III*, *blaZ, mph(C), msr(A), and sat-4*] conferring resistance to aminoglycosides, beta-lactams, macrolides, and nucleosides (Fig. 7A). An-SA displayed two prevalent plasmid/multi-ARGs associations (Fig. 7B). The first involved seven ARGs, *aac(6’)-aph(2’’*), *aadD*, *ant (6)-Ia*, *erm(C*), *lnu(B*), *lsa(E*), and *tet(L*), carried by a Rep1 plasmid and conferred resistance to aminoglycosides, macrolides, lincosamides, and tetracyclines (42/244). The second involved the *aadD*, *tet(L*), and *tet(T*) genes carried by an Inc18/Rep1 plasmid (*n* = 13).

#### Transposon/ARG associations

Apart from *erm(A*), *ant (9)-Ia,* and *erm(B*) that were each found on two different transposons, all other ARGs were associated with one type of transposon, and identical transposon/ARG associations were identified in both human and animal genomes (Fig. 6). The Tn*554/ant (9)-Ia*, Tn*554*/*erm(A*), and Tn*558*/*fexA* associations were the most frequently detected in Hm-SA and An-SA, respectively (Fig. 6). Some ARGs, such as *blaZ*, were also found associated with both transposons and ICEs (Table S3). A strong association between transposons and genes conferring resistance to lincosamide or phenicol (such as *lnu(G*), *vga(A)V*, *vga(E*), or *fexA*) was observed (Fig. 6). Transposons also harbored multiple ARGs conferring resistance to one or two antibiotic families. Notably, the Tn*554*/*erm(A*)/*ant (9)-Ia* association emerged as the most frequent MGE/multi-ARGs association in Hm-SA (3,643/9,241) and the second most common in An-SA (25/320) (Fig. 7). Additionally, the Tn*6133*/*erm(A*)/*ant (9)-Ia*/*vga(E*) association was identified in both Hm-SA (25/3,668 transposon/multi-ARGs associations) and An-SA (11/36) genomes (Fig. 7).

#### Integrative and conjugative element/ARG associations

Overall, a minority (<9%) in both Hm-SA (1,430/40,911 ARG associated) and An-SA (270/3,266) of ARGs were associated with ICEs, but 20 (including seven in An-SA) different associations were identified (Table S3). The most prevalent element was Tn*916*/*tet(M)* (Fig. 6). The *tet(M*) gene was usually located on ICEs (1,076/1,126 *tet(M*) gene identified in Hm-SA and 246/246 in An-SA) (Fig. 6) and sometimes associated with other resistance genes like *dfrG* in Hm-SA and An-SA, or *blaZ* and *mecA* in Hm-SA (Fig. 7).

#### Staphylococcal chromosomal cassette/ARG associations

The *mecA* gene was carried by several types of SCC*mec* cassettes, while *mecC* was exclusively associated with the type XI(8E) element. In addition to *mec* genes, 23 different ARGs were found on SCC*mec* elements and formed 97 SCC*mec*/ARG associations in Hm-SA genomes, while nine different ARGs were found forming 12 different SCC*mec*/ARG associations in An-SA strains (Table S3). In Hm-SA, the most common genes were *ant (9)-Ia* and *erm(A*) genes carried by a type II(2A) SCC*mec* element [1,513/4,099 SCC*mec*/ARG associations excluding *mecA* and *mecC*, for *ant (9)-Ia* and 1,473/4,099 for *erm(A*)]. In An-SA genomes the type XI(8E) cassette carrying *mecC* was always associated with *blaZ* (10/40 SCC*mec*/ARG associations excluding *mecA* and *mecC*) (Table S3). The SCC*mec*/multi-ARGs associations, like type Vc/*mecA*/*tet(K*) and type IVa/*mecA*/*dfrC*, were found in both humans and animals even though their proportions differed depending on the origin (*n* = 27 and 41/1,961 SCC*mec*/multi-ARGs associations in Hm-SA, *n* = 8 and 3/27 in An-SA) (Fig. 7; Table S3).

#### Prophage/ARG associations

Only 0.3% of ARGs of human origin (160/40,911 ARGs associated) and 0.1% of animal origin (4/3,266) were found associated with prophages. In Hm-SA genomes, the most common prophage/ARG associations were phi2958PVL/*dfrG* (27/160 prophage/ARG associations) and SPbeta_like/*aac(6')-aph(2'')* (23/160) (Table S3). This element was found associated with 17 different ARGs contributing to resistance to eight antibiotic families (Table S3). Prophage/ARG associations were not exclusive, i.e., *blaZ* could be associated with 10 different types of prophages. In An-SA genomes, only two associations were identified, i.e., *aac(6')-aph(2''*)/*aph(3')-III*/*ant (6)-Ia* carried by a SPbeta-like prophage, and *dfrG* associated with YMC/09/04/R1988.

#### Composite transposon/ARG associations

These MGEs were only associated with about 5% of ARGs in both Hm-SA (2,510/40,911 ARGs associated) and An-SA (190/3,266) (Table S3). IS*Sau6* was the most common CTn, associated with several ARGs conferring resistance to all antibiotic families identified in *S. aureus* genomes (24 different ARGs in Hm-SA and 15 in An-SA genomes) (Table S3). In Hm-SA, the most frequently identified associations were IS*Sau6*/*aadD* (547/2,510 CTn/ARG associations), and IS*Sau6*/*mecA* (*n* = 534), this association is also the most abundant in An-SA (103/190). Of note, these associations were both located on a SCC*mec* element.

## DISCUSSION

Although MGEs are recognized key players in the dissemination of AMR, the association between MGEs and ARGs has been understudied in Gram-positive bacteria, particularly in *S. aureus*. While previous studies focused either on MGEs and/or ARGs (8, 9, 20, 21), our global-scale analysis of over 10,000 publicly available *S. aureus* genomes from both animal and human origin is, to our knowledge, the first to reveal the intricate interplay between mobilome and resistome. Our analysis identified 433 STs, 278 MGEs, and 78 different ARGs in *S. aureus*. Their associations highlighted not only the expected clonal expansion but also significant horizontal transfer events, underscoring HGT as a key driver in the evolution of *S. aureus* populations.

Our study, like all studies based on publicly available genomes, has limitations imposed by the bias in sequenced genomes deposited in the NCBI database. Due to sequencing costs, only strains harboring the most critical resistance genes for human and animal health were analyzed. This resulted in an over-representation of MRSA strains (80% in Hm-SA and 71% in An-SA) and the under-representation of An-SA, except for *mecA*-positive ST398 and ST9 isolates of porcine origin (22%) and MRSA of bovine origin (12%). However, since our study aimed to capture the different types of MGEs and ARGs rather than quantify them, this bias should only mildly interfere with our analyses. Moreover, analyzing >10,000 genomes from diverse geographical locations, and from more than 16 different hosts, should help to mitigate this bias.

Our study revealed the presence of 50 different ARGs in *S. aureus* of human and animal origin, an even larger number than the one (*n* = 46) reported in the review by Schwartz et al. (7), capable of conferring resistance to most antibiotic families, including those considered as CIA for humans, animals, or for both (such as third and fourth generation cephalosporins or macrolides) (18, 19). The most abundant genes were *blaZ* and *mecA*, as also pointed out in a recent study of AMR in publicly available *S. aureus* genomes (22). A recent study on the resistome/mobilome association in ESKAPE pathogens highlighted the overall high prevalence of genes conferring resistance to aminoglycosides, chloramphenicol, trimethoprim, and tetracyclines (23). Genes conferring resistance to these antibiotic families were also abundant (18/44 ARGs identified) in our study dedicated to *S. aureus*, and their dissemination is consistent with antibiotic use in both human (24) and veterinary medicine (25). Globally, our study revealed a higher number of different ARGs in Hm-SA and a larger set of human-specific genes compared to those reported in ESKAPE pathogens (23). This could be explained by the larger number of genomes analyzed, but also by the higher exposure of humans to antibiotic selection in various environments (hospitals, farm environments). Nevertheless, multiple resistance genes were also found in animals, including in pigs that are known to be asymptomatic carriers of *S. aureus*. In these animals, the acquisition of resistance can be selected by treatments for non-staphylococcal infections, making them reservoirs and a breeding ground for HGT. Only one gene, *optrA*, was specific to An-SA. This gene, first discovered in human and animal enterococci on a conjugative plasmid (26), and then observed in *Staphylococcus sciuri* of porcine origin (27), is conferring resistance to phenicols. It was found on the *S. aureus* chromosome near the SCC*mec*, but also on a plasmid with the *cfr* gene (27). In our study, *optrA* was identified on the chromosome and not associated with an MGE: nevertheless, it was located <10 kb from the *fexA* gene carried by Tn*558*, an MGE/ARG association already found on the plasmid carrying *cfr* in other staphylococci (28, 29). Besides these host specificities, a large set of genes, among which *aph(3’)-III*, *dfrC*, *fusA*, *tet(T*), *apmA,* and *vga(E*), was shared between An-SA and Hm-SA. This suggests that numerous ARGs have no host boundaries and can be disseminated from human *S. aureus* to animals and vice versa.

The *S. aureus* mobilome was largely composed of IS and phages, which rarely carried ARGs, followed by plasmids and transposons that were identified as the main carriers of ARGs for this species. Our study also highlighted the high diversity of *S. aureus* plasmids, with 297 different *rep* gene combinations often forming mosaic plasmids (12, 30) with multiple *rep* genes (37% of plasmids in Hm-SA and 23% in An-SA). Previous studies mainly reported the association of only two *rep* genes, belonging to the same or two different plasmid families (31, 32). However, our work demonstrated that as many as eight different *rep* genes can be found in a single plasmid. Unlike plasmids circulating in Enterobacterales, those identified in *S. aureus* present few Rep protein incompatibilities. This facilitates the formation of mosaic plasmids, enabling *S. aureus* to adapt more effectively to multiple hosts and environmental conditions (31, 33–35). Furthermore, the presence of numerous insertion sequences (IS elements) and the rolling-circle replication system of small plasmids (<10 kb) favors the recombination and fusion of several small plasmids (31), resulting in unique large hybrids carrying multiple *rep* genes.

The prevailing view for a long time held that *S. aureus* possessed very few plasmids and horizontal gene transfer was believed to occur primarily through transduction, so only a few studies focused on the mechanism of transfer of plasmids in *S. aureus*. Literature in Gram-positive bacteria showed that plasmids can be mobilized by using the conjugation system of other plasmids or ICEs present in the strain (15, 36–38). A study in *Bacillus subtilis* showed that plasmids can be mobilized by the *ori*T of ICE*Bs1,* an ICE of the Tn*916* family (37, 38). In *S. aureus*, plasmids can be mobilized if they possess a relaxase or by forming co-integrates that are subsequently resolved by “conduction” (39, 40). O'Brien et al. (41) showed that the conjugative plasmid pWBG749 could mobilize the transfer of numerous small plasmids carrying an *ori*T similar to its own (41, 42). In line with our results that showed the importance of plasmids as ARG carriers, further studies are now needed to understand the mechanisms by which these plasmids spread and to ultimately limit their propagation.

Often misclassified in the literature as transposons or plasmids (43), ICEs are also capable of HGT. Studies on enterococci and streptococci showed that these MGEs play a crucial role in the dissemination of ARGs (44). The well-known Tn*916* ICE family was found in many Firmicutes, always carrying the *tet(M*) gene (45–47). This element exhibits a similar backbone sequence between *S. aureus* and streptococci (46). Within *S. aureus*, it was first described in the Mu50 strain and named Tn*5801* (48). Our study identified another ICE, ICE*6013*, as the most prevalent family typically harboring few resistance genes, but capable of carrying other MGEs like Tn*552*, often associated with the *blaZ gene*. This association of multiple MGEs with ARG was first identified in *S. aureus* ST239 (14). Despite sharing some protein similarities with the ICE*Bs1* of *B. subtilis*, ICE*6013* belongs to a distinct family (14). Other MGEs were also found nested within each other, like transposons found within plasmids and SCC*mec* cassettes. For instance, Tn*554*/*ant (9)-Ia*/*erm(A*) was found on both Rep1 plasmids and SCC*mec* type II(2A) cassettes. The interweaving of MGEs significantly amplifies the potential for dissemination of ARGs (14, 43).

Beyond revealing the diversity of ARGs and MGEs in *S. aureus*, our study importantly highlighted numerous associations between these elements. The high degree of similarities between MGE/ARG associations observed in An-SA and Hm-SA strongly suggested unrestricted dissemination of MGEs across hosts. This finding implies that An-SA could serve as a reservoir for Hm-SA and vice versa. Our results identified a few dominant STs within Hm-SA or An-SA populations, typically harboring the same set of ARG/MGE associations. This observation highlights clonal expansion as a major driver of dissemination in *S. aureus*. This is the case for *S. aureus* ST22, initially identified in United Kingdom hospitals (49), which has become a globally disseminated epidemic clone and has spread across Europe and worldwide, impacting both healthcare settings and the community, including domestic animals like dogs and cats (50, 51). Due to this pandemic character, very few variations were observed in ST22 MGE content in genomes available in public databases. Although our study did not capture evidence of such events, the reported presence of a plasmid carrying via Tn*558* the *cfr* and *fexA* genes in Irish ST22 isolates (29) demonstrates the potential for horizontal transfer even within this otherwise stable clonal lineage. Besides this specific example of a rare event, our results also evidenced more frequent transfers. Notably, within the same ST, the observed diversity of MGEs and ARGs demonstrates that the mobilome and resistome are sources of significant genomic plasticity, despite core genome similarity. Furthermore, our analysis revealed an intense spread of certain MGE/ARG associations across diverse STs (Fig. 6). For instance, the Rep1/*aadD* association was found in all five most prevalent STs. Additionally, the Tn*916*/*tet(M*) was primarily associated with ST398 in An-SA (80% of identified *tet(M*) genes are in ST398), while in Hm-SA, it was found in both ST398 (40%) and ST239 (20%). This suggests potential host adaptation, in line with recent studies demonstrating the role of *tet(M*)-carrying Tn*916* transposon, facilitating the jump from Hm-SA to An-SA (52). Despite lacking their transfer systems and residing primarily within chromosomes, transposons also contribute to *S. aureus* genome plasticity and resistome dissemination among STs. Our findings revealed the presence of transposons in diverse STs, like Tn*554*/*ant (9)-Ia*/*erm(A*) which was identified in more than 12 STs, and Tn*558*/*fexA* found in 10 An-SA STs. This widespread distribution across different epidemic clones highlights the horizontal propagation of these elements, suggesting their important role in shaping the resistome and, more broadly the genomes of *S. aureus*. Our results point out that antibiotic selection (through treatments, but also metaphylaxis or residues depending on the host or the environment) can promote the dissemination of these elements, as well as co-select resistance when multiple genes are located on the same genetic determinant.

In conclusion, this study revealed the remarkable abundance and diversity of MGEs and ARGs within *S. aureus* genomes, primarily associated with plasmids and transposons. While it is known that *S. aureus* is disseminated through clonal waves, our results showed that clones can also evolve through horizontal acquisition of MGE/ARG associations that promote the dissemination of resistance genes without any specificity for a particular antibiotic family. This study provides additional evidence that An-SA can serve as reservoirs for Hm-SA and vice versa and that the mobilome’s only limitation is its transfer efficiency. Plasmids in *S. aureus*, as primary reservoirs of ARGs, are key players in AMR dissemination. These MGEs carry many antibiotic-resistance genes, notably those conferring resistance to human and animal CIAs defined by the WHO and WOAH. All these findings highlight the critical need to elucidate the mechanisms governing the epidemic success of MGEs, particularly those implicated in ARG transfer.

## MATERIALS AND METHODS

### Genome characterization and phylogenetic analysis

Genome assemblies (complete and draft genomes) of *S. aureus* of animal (*n* = 1,436) and human origin (*n* = 11,888) were downloaded from the NCBI public database in October 2021. Quality and completeness of assemblies were evaluated using QUAST v.5.0.2 (53) and BUSCO v.5.2.1 (54) (Fig. S5). All contigs <1,000 bp were removed from the analysis. Genomes were characterized according to their ST using MLST v.2.1.1 (55). Transposons, ICE, IS, and CTn were identified using MobileElementFinder v.1.0.3 (80% coverage and 70% identity) (56). Plasmid replicons (*rep* coding gene) were detected using PlasmidFinder v.2.1.6 software (databases: v.2021-11-29) (60% coverage and 90% identity) (57, 58). SCCs were identified using SCC*mec*Finder v.1.2.1 (database extended, 60% coverage, and 90% identity) (57, 59, 60). ARGs were identified using ResFinder v.4.1.7 (database: v.2021-09-22) (60% coverage and 80% identity) (57, 61) and RGI-CARD (Comprehensive Antibiotic Resistance Database) with a percentage length of the reference sequence <200 (62). Co-occurrence was considered when several ARGs were identified within the same strain. A phylogenetic tree was constructed using pyMLST v.2.1.3 with default parameters (63). A neighbor-joining tree based on the *S. aureus* core genome was constructed using cgMLST software (https://www.cgmlst.org/ncs) (64) based on a list of core genes (Table S2). To avoid duplicates, only one genome with identical MGE and ARG content, geographical origin, collection year, and NCBI bioProject was selected in each group determined by cgMLST with a maximum distance of 10 different alleles (Fig. S5). Following this selection, a total of 9,408 genomes of Hm-SA and 655 genomes of An-SA, carrying at least one ARG and one MGE (except IS) were included in this study.

### Identification and characterization of detected mobile genetic elements

#### Plasmids identification

PlasForest (57, 65, 66), RFPlasmid for *Staphylococcus* species (67), Plasmer (68), and Plasflow (69) were used with default parameters to identify whether contigs were plasmidic or chromosomal. A contig was considered plasmidic if predicted by at least three software. Due to the fragmented nature of draft genomes, some plasmids were split into multiple contigs during assembly. To identify contigs belonging to the same plasmid, those identified as plasmidic were analyzed using BLASTN. Contigs were considered part of the same plasmid if they shared more than 60% identity and coverage with the same reference plasmid sequence. Some previously unclassified contigs were identified as plasmidic due to their similarity to known plasmids. Plasmids were classified according to the active protein domain of the Rep protein that was encoded by the *rep* gene. Since only the potential of dissemination of ARGs was evaluated, all *rep* genes identified on chromosomal contigs were excluded from the analysis (381/1,608; 24% *rep* genes identified in An-SA; and 4,240/22,966; 28% in Hm-SA) (Table S4). These chromosomal *rep* genes have been identified as associated with ARG already carried by other MGEs (ICE, SCC*mec*, phage) in An-SA and not associated with ARG (i.e., ARG found within 30 kb around the *rep* genes) in Hm-SA (Table S4).

#### Composite transposons identification

A CTn was predicted when two IS were at a specified distance from each other. The threshold value was 12 kb according to the length of the largest CTn identified in *S. aureus* (8).

#### Staphylococcal chromosomal cassette identification

Boundaries of SCC were fixed with *orfX* gene and the end was adjusted based on the size of the cassette type (https://www.sccmec.org/). SCCs found scattered across multiple contigs were adjusted based on gene order (synteny) to define their limits. SCC*mec*Finder v.1.2.1 did not determine the cassette type for some SCC elements, considered as “indeterminate.” SCC lacking *mecA* or *mecC* genes were classified as “SCC” instead of SCC*mec*.

#### Prophages identification

These elements were identified using PHASTEST, last accessed October 2023 (70–72). Only prophages with a “complete” score were retained. Prophages were named according to the bacteriophage exhibiting the closest similarity, determined by both sequence identity, and the number of common genes.

#### Integrative and conjugative elements identification

In addition to MobileElementFinder v.1.0.3 detection, a BLASTP analysis was performed against a custom database created from literature and ICEBerg 2.0 database (15, 48, 73, 74). This database included conserved components of the two main ICE families, Tn*916* and ICE*6013*, identified in a previous study (73) (Fig. S6A). Only conserved elements meeting the following criteria were considered as ICEs: proximity between elements, number of conserved elements on the same contig, and integrity of the gene (Fig. S6B). ICE boundaries were extended by 10 kb at each end of the ICE, reflecting the average size of Staphylococci ICE (approximately 30 kb).

### Identification of associations between antibiotic resistance genes and mobile genetic elements

To assess the correlation between the number of ARGs and the number of each MGE within a genome, the Pearson correlation coefficient was calculated using GraphPad Prism 9.5.1 (GraphPad Software Inc., CA, USA). MGE/ARG associations were identified through co-localization analysis when the MGE region entirely overlapped the ARG region. This rule was applied only for transposons, ICE, SCC, prophages, and CTn. For plasmids, an ARG was considered associated when it was localized on a plasmidic contig (see above). For plasmidic contig lacking *rep* gene, associated ARGs were considered carried by a plasmid of a non-determined type. Unclassified contigs identified as associated with SCC, prophage, and ICE were reclassified as chromosomal. When multiple ARGs were found within the same MGE from a single strain, the MGE was considered to harbor multiple ARGs.

## SUPPLEMENTAL MATERIAL

Table S1 mbio.02428-24-s0001.xlsx Database of MGEs and ARGs detected in *S. aureus* of human and animal origin.

Table S2 mbio.02428-24-s0002.xlsx List of core genes of *S. aureus* of human and animal origin.

Table S3 mbio.02428-24-s0003.xlsx Database of MGE/ARG associations in *S. aureus* of human and animal origin.

Supplemental material mbio.02428-24-s0004.pdf Supplemental figures, Table S4, and legends for supplemental tables.

ASM does not own the copyrights to Supplemental Material that may be linked to, or accessed through, an article. The authors have granted ASM a non-exclusive, world-wide license to publish the Supplemental Material files. Please contact the corresponding author directly for reuse.
