# Supplementary material for: The interplay between mobilome and resistome in Staphylococcus aureus
Source: mBio. 2024 Sep 17;15(10):e02428-24. doi: 10.1128/mbio.02428-24 (PMC11481524; doi:10.1128/mbio.02428-24)
Supplement: Supplemental material — Supplemental figures, Table S4, and legends for supplemental tables. [file mbio.02428-24-s0004.pdf]

Supplementary data

Table S1. Database of MGEs and ARGs detected in *S. aureus* of human and animal origin.

Table S2. List of core genes of *S. aureus* of human and animal origin.

Table S3. Database of MGE/ARG associations in *S. aureus* of human and animal origin.

Table S4. Chromosomal *rep* gene identified and excluded from the analysis in *S. aureus* genomes of human (A) and animal (B) origin.

Figure S1. Occurrence and diversity of sequence types (STs) in *S. aureus* according to their host (A) and depending on human (Hm-SA, black) and animal (An-SA, white) origin (B). (A) The frequency of each ST corresponds to the number of strains classified by their respective ST and host, divided by the total number of strains of that ST. Each host corresponds to a color as defined on the right of the graph. Hosts grouped under the label 'others' correspond to dolphins (n=4), bats (n=4), fishes (n=2), cervid (n=1), shrimp (n=1) and monkey (n=1). Only STs with a frequency of at least >1% in Hm-SA or An-SA, were represented. **(B)** The frequencies corresponded to the number of genomes of each type divided by the total number of genomes according to their source (Hm-SA n=9,408 and An-SA n=655).

Figure S2. Frequency of mobile genetic elements (MGEs) and antibiotic resistance genes (ARGs) depending on the geographical location in *S. aureus* of human (Hm-SA) (A&C) and animal (An-SA) (B&D) origin. The frequencies of ARGs and MGEs were calculated based on the number of genomes carrying each ARG or MGE within their respective geographical location, divided by the total number of genomes carrying each ARG or MGE. Only ARGs with a frequency of at least >0.1% in Hm-SA or in An-SA, were represented. Highlighted genes are categorized as follows (i) those conferring resistance to antibiotics listed as "human-use only", HICIA and CIA in the WHO list (green), (ii) those listed as

veterinary CIA in the WOAH list (brown), and (iii) those included in both lists (blue). Above each bar, the proportion (in %) of genomes carrying each ST (A & B) and GRA (C & D) has been added. The asterisk in C indicated a proportion of 0.04%.

Figure S3. Frequency of mobile genetic elements (MGEs) and antibiotic resistance genes (ARGs) depending on sequence types (STs) in *S. aureus* of human (Hm-SA) (A&C) and animal (An-SA) (B&D) origin. The frequencies of ARGs and MGEs were calculated based on the number of genomes carrying each ARG or MGE within their respective ST, divided by the total number of genomes carrying each ARG or MGE. Only ARGs with a frequency of at least >0.1% in Hm-SA or in An-SA, were represented. Highlighted genes are categorized as follows (i) those conferring resistance to antibiotics listed as "human-use only", HICIA and CIA in the WHO list (green), (ii) those listed as veterinary CIA in the WOAH list (brown), and (iii) those included in both lists (blue). Above each bar, the proportion (in %) of genomes carrying each ST (A &B) and GRA (C &D) has been added. The asterisk in C indicated a proportion of 0.04%.

Figure S4. Association between antibiotic resistance genes (ARGs) in *S. aureus* of human (Hm-SA) (A) and animal (An-SA) (B) origin. Links indicated that the two genes were found within the same genome. The thickness of the line was proportional to the amount of genomes carrying this co-occurrence. The thickest lines correspond to *blaZ/mecA* association identified in 417 An-SA genomes and 5,913 Hm-SA genomes. Only ARGs found in >1% of Hm-SA or An-SA genomes were represented. Each node is colored according to the ARG antibiotic family. 'TMP' corresponds to trimethoprim, 'Others' to nucleoside (*sat-4)* and mupirocin (*mupA)* antibiotic families.

Figure S5. Selection of *S. aureus* genome of human (Hm-SA) and animal (An-SA) origin. This decision tree shows the process that was followed to select the genomes used in our study. One genome with only a large amount of insertion sequence (n=87) (GCA_014876755.1) (a), and one with incoherent cgMLST profile (GCA_001921685.1) (b) were excluded. (c) Six genomes for which not all analyses were achievable with NCBI data, due to the unavailability of certain files, were also excluded (GCA_014337115.1; GCA_014336505.1; GCA_014353635.1; GCA_014353595.1; GCA_014353515.1; GCA_900018155.1). MGE: mobile genetic element; ARG: antibiotic resistance genes IS: Insertion Sequence; Tn: transposon; ICE: Integrative and Conjugative Element; SCC: Staphylococcal Chromosomal Cassette; CTn: composite transposon.

Figure S6. Criteria for integrative and conjugative elements (ICEs) identification and validation after BLASTP analyses. (A) List of proteins or protein domains used to identify the presence of one of the two ICE families. **(B)** Decision tree showing how the presence or absence of an ICE has been validated.

Table S4. Chromosomal *rep* gene identified and excluded from the analysis in *S. aureus* genomes of human (A) and animal (B) origin.

A

Rep families	SCC*mec*/ARG	ICE/ARG	Prophage/ARG	No MGE/ARG	No MGE/No ARG	Total
Enterobacterales					6	6
Inc18	23		2	38	120	183
Rep_trans	80	607	4	9	2,367	3,064
Rep1	549	7	6	78	11	651
Rep2				1		1
Rep3	2		1	15	101	119
RepA_N	33	1	6	35	140	213
RepL	1			2		3

B

Rep families	SCC*mec*/ARG	ICE/ARG	Prophage/ARG	No MGE/ARG	No MGE/No ARG	Total
Inc18					6	6
Rep_trans	8	209			77	294
Rep1	2			69		71
Rep3					1	1
RepA_N				1	8	9

Each number in the first three columns represent the count of *rep* genes identified on a chromosomal contig associated with an ARG-carrying MGE (SCC*mec*/ARG, ICE/ARG and Prophage/ARG). The "no MGE/ARG" column corresponds to the number of chromosomal *rep* gene that are associated with ARGs (i.e 30 kb around *rep* gene) not associated with an MGE. The "no MGE/ no ARG" column corresponds to chromosomal *rep* gene not that are neither associated with an ARG-carrying MGE nor linked to an ARG. The last column is the total number of *rep* genes identified on a chromosomal contig.

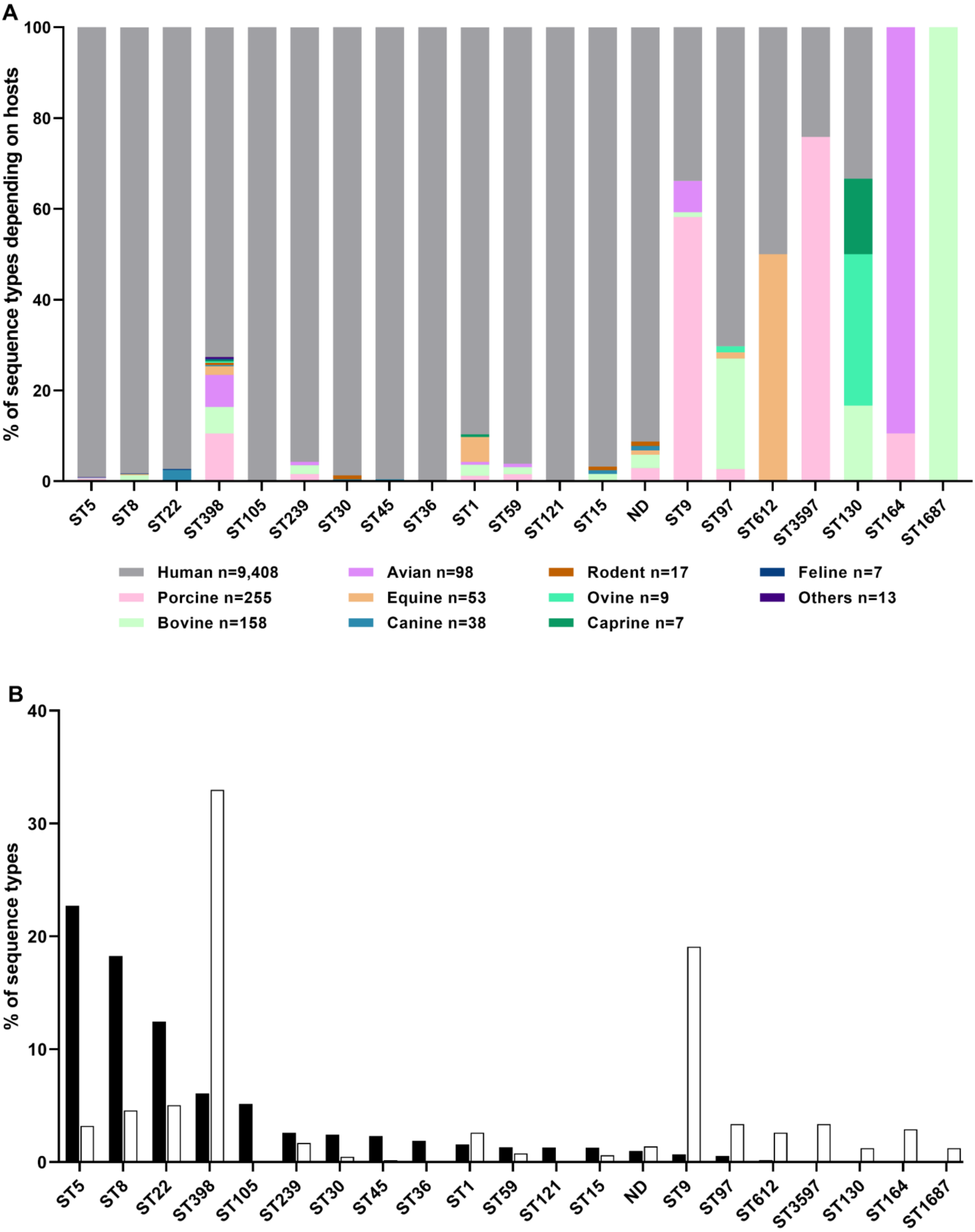
A
B
% of sequence types depending on hosts
% of sequence types
ST5
ST8
ST22
ST398
ST105
ST239
ST30
ST45
ST36
ST1
ST59
ST121
ST15
ND
ST9
ST97
ST612
ST3597
ST130
ST164
ST1687
Human n=9,408
Porcine n=255
Bovine n=158
Avian n=98
Equine n=53
Canine n=38
Rodent n=17
Ovine n=9
Caprine n=7
Feline n=7
Others n=13

Figure S1.

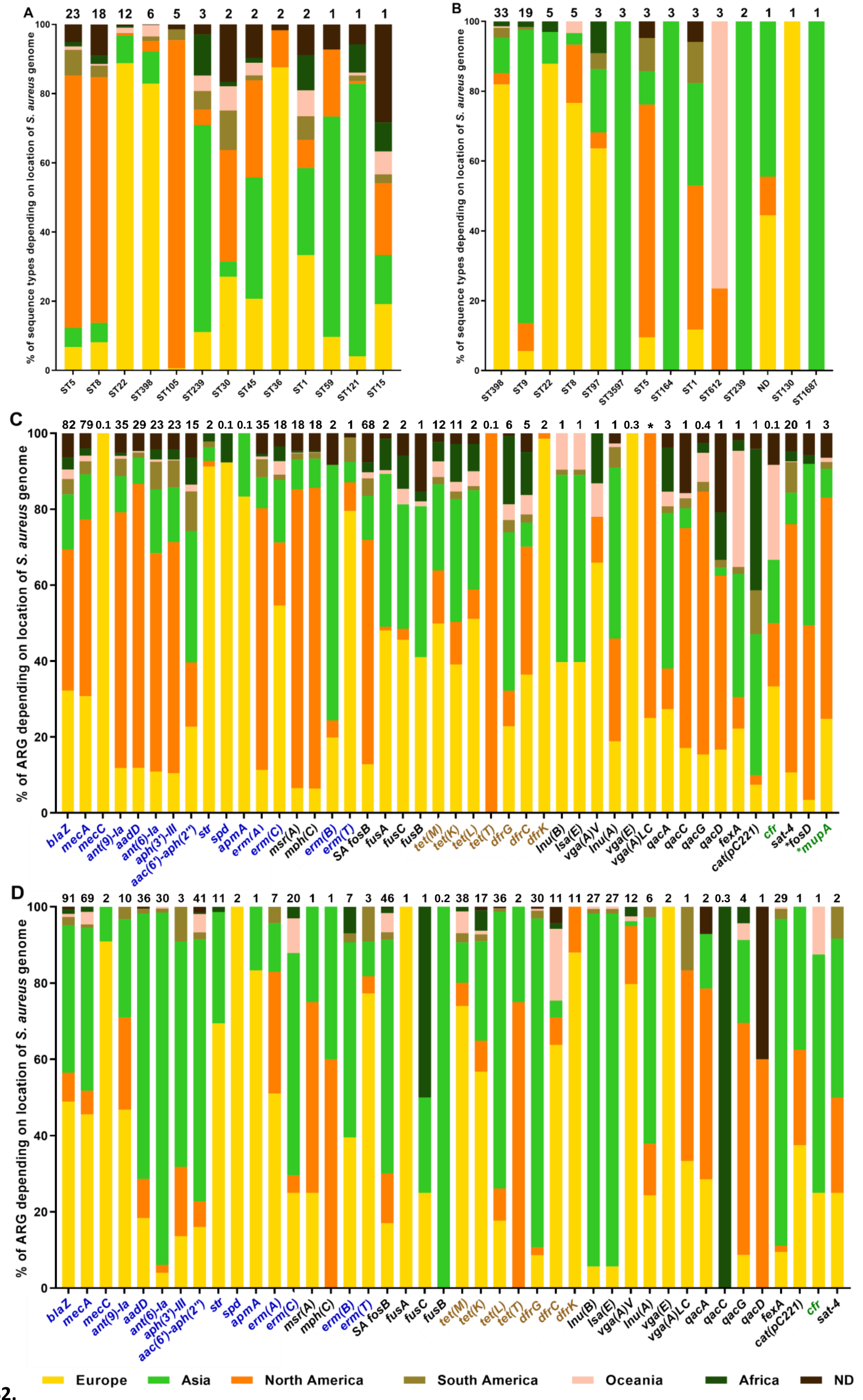
A
B
C
D
% of sequence types depending on location of S. aureus genome
% of ARG depending on location of S. aureus genome
ST5 ST8 ST22 ST398 ST105 ST239 ST30 ST45 ST36 ST1 ST59 ST121 ST15
ST398 ST9 ST22 ST8 ST97 ST3597 ST5 ST164 ST1 ST612 ST239 ND ST130 ST1687
blaZ mecA mecC ant(9)-Ia aadD ant(6)-Ia aph(3')-III aac(6')-aph(2'') str spd apmA erm(A) erm(C) msr(A) mph(C) erm(B) erm(T) SA fosB fusA fusC fusB tet(M) tet(K) tet(L) tet(T) dfrG dfrC dfrK lnu(B) lsa(E) vga(A)V lnu(A) vga(E) vga(A)LC qacA qacC qacG qacD fexA cat(pC221) cfr sat-4 *fosD *mupA
Europe
Asia
North America
South America
Oceania
Africa
ND

Figure S2.

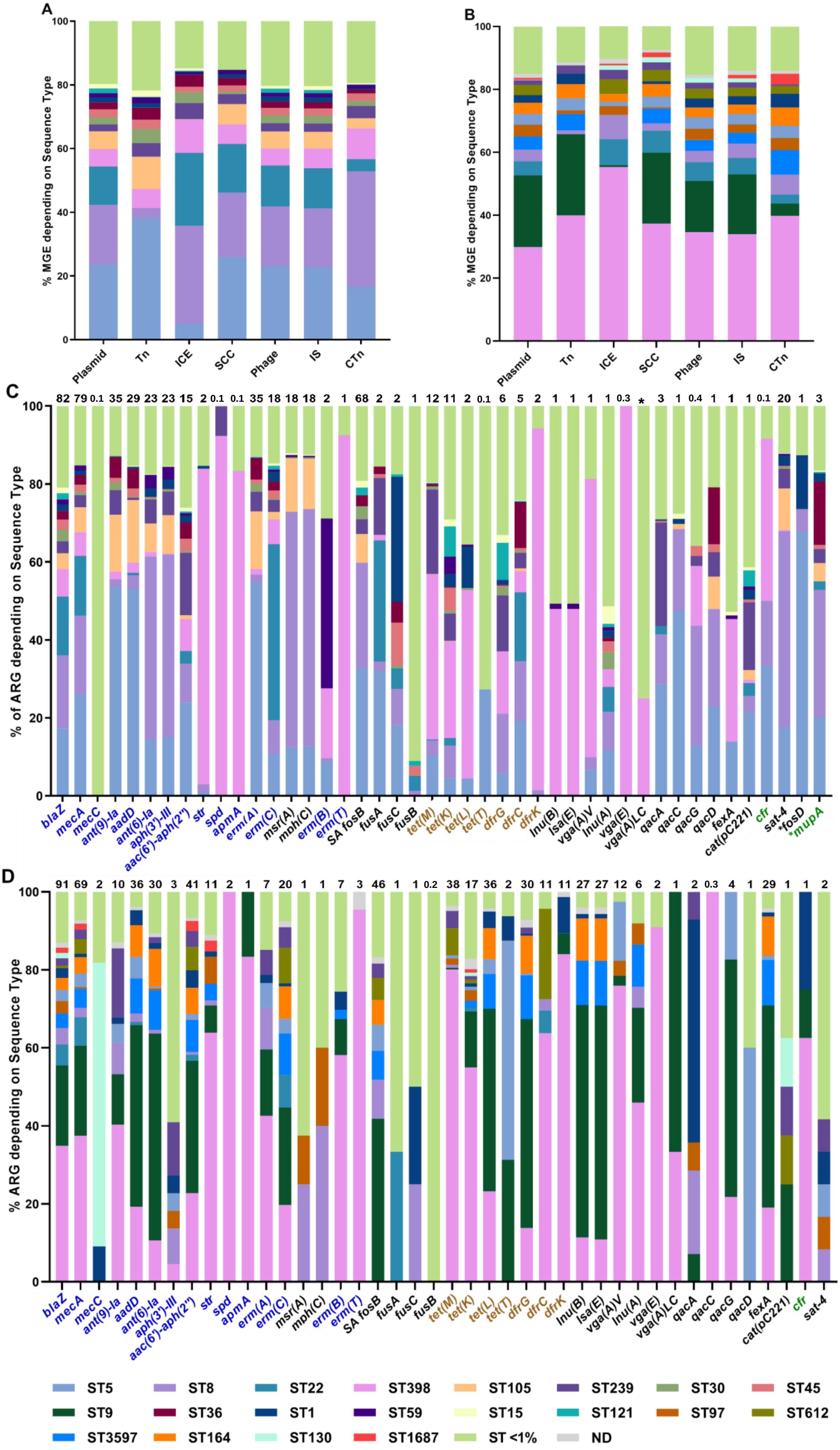

A
B
% MGE depending on Sequence Type
0
20
40
60
80
100
Plasmid
Tn
ICE
SCC
Phage
IS
CTn
C
% of ARG depending on Sequence Type
82 79 0.1 35 29 23 23 15 2 0.1 0.1 35 18 18 18 2 1 68 2 2 1 12 11 2 0.1 6 5 2 1 1 1 1 0.3 * 3 1 0.4 1 1 1 0.1 20 1 3
blaZ mecA mecC ant(9)-Ia aadD ant(6)-Ia aph(3')-III aac(6')-aph(2'') str spd apmA erm(A) erm(C) msr(A) mph(C) erm(B) erm(T) SA fosB fusA fusC fusB tet(M) tet(K) tet(L) tet(T) dfrG dfrC dfrK lnu(B) lsa(E) vga(A)V lnu(A) vga(E) vga(A)LC qacA qacC qacG qacD fexA cat(pC221) cfr sat-4 *fosD *mupA
D
% ARG depending on Sequence Type
91 69 2 10 36 30 3 41 11 2 1 7 20 1 1 7 3 46 1 1 0.2 38 17 36 2 30 11 11 27 27 12 6 2 1 2 0.3 4 1 29 1 1 2
blaZ mecA mecC ant(9)-Ia aadD ant(6)-Ia aph(3')-III aac(6')-aph(2'') str spd apmA erm(A) erm(C) msr(A) mph(C) erm(B) erm(T) SA fosB fusA fusC fusB tet(M) tet(K) tet(L) tet(T) dfrG dfrC dfrK lnu(B) lsa(E) vga(A)V lnu(A) vga(E) vga(A)LC qacA qacC qacG qacD fexA cat(pC221) cfr sat-4
ST5 ST8 ST22 ST398 ST105 ST239 ST30 ST45
ST9 ST36 ST1 ST59 ST15 ST121 ST97 ST612
ST3597 ST164 ST130 ST1687 ST <1% ND

Figure S3.

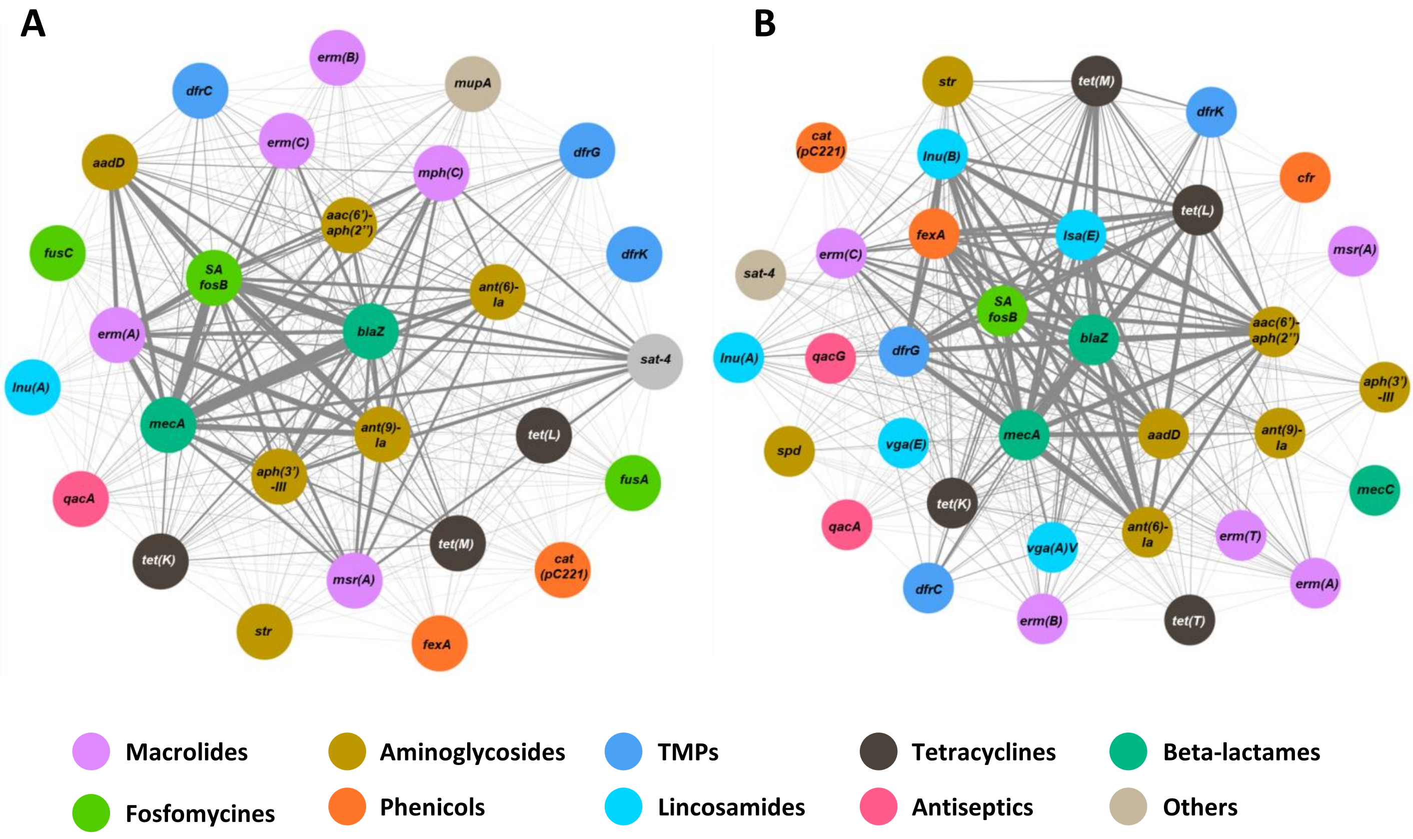
A
erm(B)
dfrC
mupA
erm(C)
aadD
mph(C)
dfrG
aac(6')-aph(2'')
fusC
SA fosB
dfrK
ant(6)-Ia
erm(A)
blaZ
sat-4
lnu(A)
mecA
tet(L)
ant(9)-Ia
aph(3')-III
fusA
qacA
tet(M)
cat (pC221)
tet(K)
msr(A)
str
fexA
B
str
tet(M)
dfrK
cat (pC221)
lnu(B)
cfr
tet(L)
fexA
lsa(E)
msr(A)
erm(C)
sat-4
SA fosB
aac(6')-aph(2'')
lnu(A)
qacG
dfrG
blaZ
aph(3')-III
mecA
aadD
ant(9)-Ia
spd
vga(E)
mecC
tet(K)
qacA
ant(6)-Ia
erm(T)
vga(A)V
erm(A)
dfrC
erm(B)
tet(T)
Macrolides
Aminoglycosides
TMPs
Tetracyclines
Beta-lactames
Fosfomycines
Phenicols
Lincosamides
Antiseptics
Others

Figure S4.

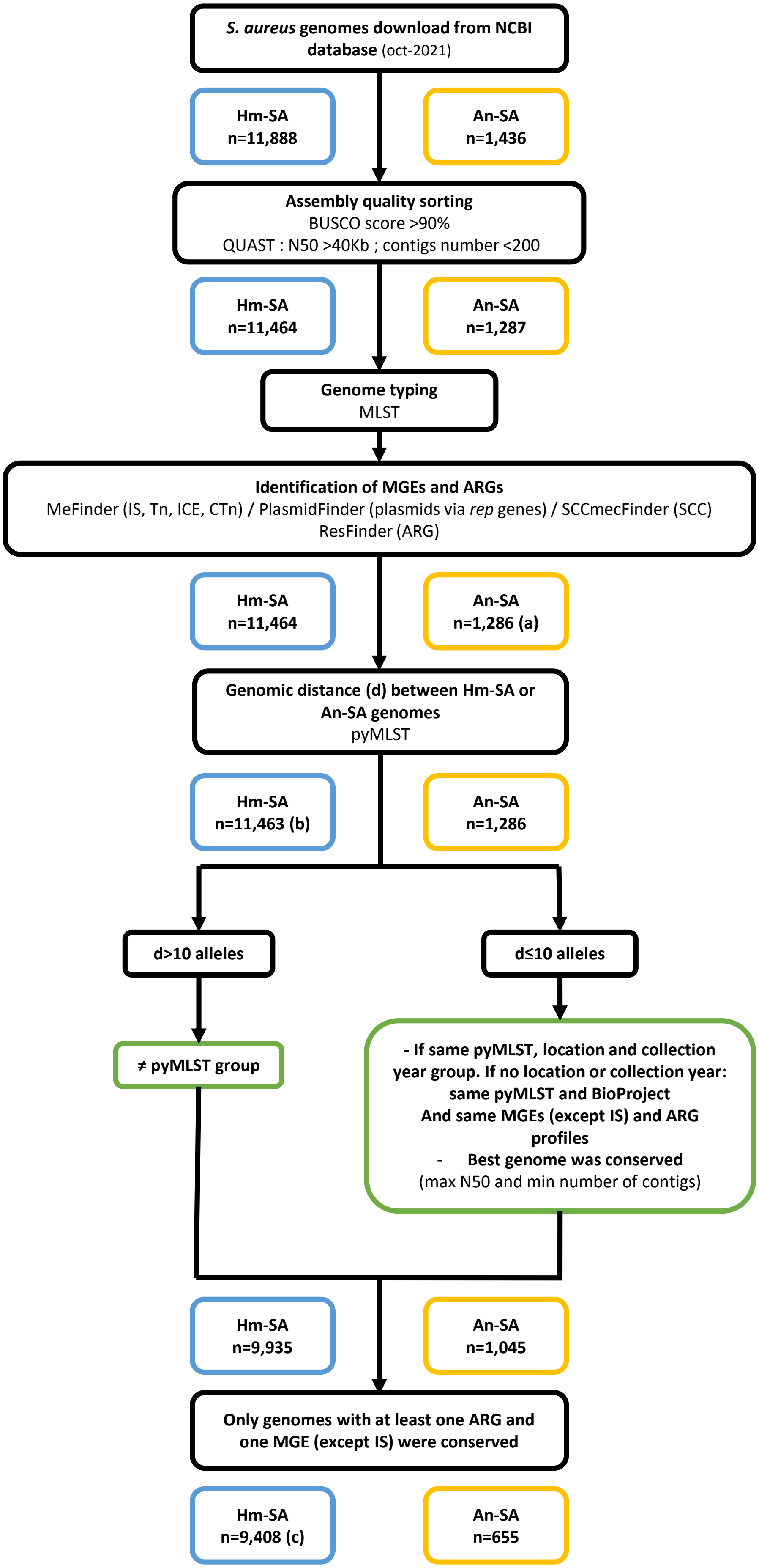
S. aureus genomes download from NCBI database (oct-2021)
Hm-SA n=11,888
An-SA n=1,436
Assembly quality sorting
BUSCO score >90%
QUAST : N50 >40Kb ; contigs number <200
Hm-SA n=11,464
An-SA n=1,287
Genome typing
MLST
Identification of MGEs and ARGs
MeFinder (IS, Tn, ICE, CTn) / PlasmidFinder (plasmids via rep genes) / SCCmecFinder (SCC)
ResFinder (ARG)
Hm-SA n=11,464
An-SA n=1,286 (a)
Genomic distance (d) between Hm-SA or An-SA genomes
pyMLST
Hm-SA n=11,463 (b)
An-SA n=1,286
d>10 alleles
d≤10 alleles
≠ pyMLST group
- If same pyMLST, location and collection year group. If no location or collection year: same pyMLST and BioProject
And same MGEs (except IS) and ARG profiles
- Best genome was conserved
(max N50 and min number of contigs)
Hm-SA n=9,935
An-SA n=1,045
Only genomes with at least one ARG and one MGE (except IS) were conserved
Hm-SA n=9,408 (c)
An-SA n=655

Figure S5.

A

ICE family	Protein Name	Protein Family	GeneBank accession number
ICE6013	YddE	VirB4 domain protein	ACI48609.1
	YdcQ	FtsK/SpoIIIE domain-containing protein	ACI48608.1
	RstA	relaxase of the MobT family	ACI48613.1
Tn916	ORF16	Conjugative transposon protein ATP-binding protein	CAQ49388.1
	ORF21	FtsK/SpoIIIE domain-containing protein	CAQ49393
	ORF22	Conjugative transposon protein YdcP family protein, DUF261	CAQ49394.1

B

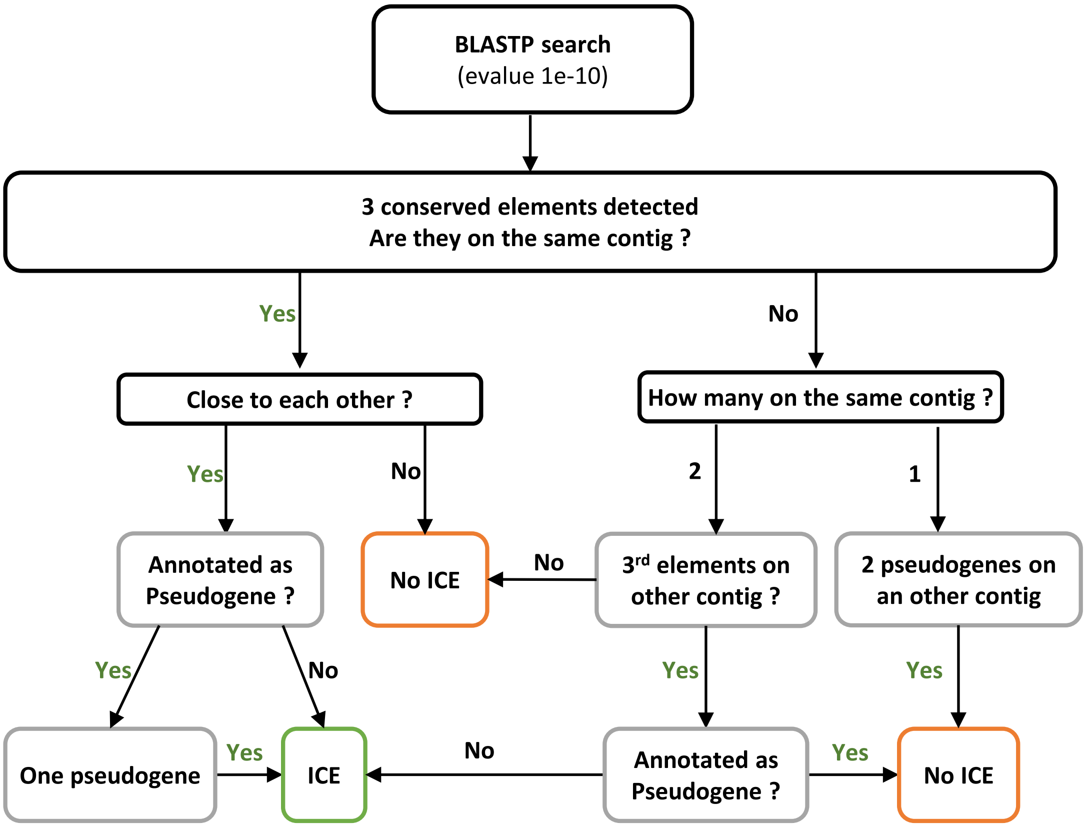

Figure S6.
